# SLy2‐overexpression impairs B‐cell development in the bone marrow and the IgG response towards pneumococcal conjugate‐vaccine

**DOI:** 10.1002/iid3.413

**Published:** 2021-02-16

**Authors:** Jennifer Jaufmann, Leyla Tümen, Sandra Beer‐Hammer

**Affiliations:** ^1^ Department of Pharmacology, Experimental Therapy and Toxicology, Institute of Experimental and Clinical Pharmacology and Pharmacogenomik and ICePhA University of Tuebingen Tuebingen Germany

**Keywords:** antibody responses, B cells, B‐cell development, pneumococcal conjugate‐vaccine, pneumonia, Src homology domain 3 lymphocyte protein 2, streptococcus pneumoniae

## Abstract

**Background:**

Infections with *Streptococcus pneumoniae* can cause severe diseases in humans including pneumonia. Although guidelines for vaccination have been established, *S. pneumoniae* is still responsible for a serious burden of disease around the globe. Currently, two pneumococcal immunizations are available, namely the pure polysaccharide vaccine Pneumovax23 (P23) and the conjugate‐vaccine Prevenar13 (PCV13). We recently reported impaired thymus‐independent antibody responses towards P23 in mice overexpressing the immunoinhibitory adapter SLy2. The purpose of this study was to evaluate adaptive B‐cell responses towards the thymus‐dependent vaccine PCV13 in SLy2‐overexpressing mice and to study their survival rate during pneumococcal lung infection. Moreover, we investigated B‐cell developmental stages within the bone marrow (BM) in the context of excessive SLy2‐expression.

**Methods:**

B‐cell subsets and their surface immune globulins were investigated by flow cytometry. For class‐switch assays, isolated splenic B cells were stimulated in vitro with lipopolysaccharide and interleukin‐4 and antibody secretion was quantified via LEGENDplex. To study PCV13‐specific responses, mice were immunized and serum antibody titers (immunoglobulin M, immunoglobulins IgG_1_, IgG_2_, and IgG_3_) were examined by enzyme‐linked immunosorbent assay. Survival rates of mice were assessed within 7 days upon intranasal challenge with *S. pneumoniae*.

**Results:**

Our data demonstrate impaired IgG_1_ and IgG_3_ antibody responses towards the pneumococcal conjugate‐vaccine PCV13 in SLy2‐overexpressing mice. This was accompanied by reduced frequencies and numbers of BM‐resident plasmablasts. In addition, we found drastically reduced counts of B‐cell precursors in the BM of SLy2‐Tg mice. The survival rate upon intranasal challenge with *S. pneumoniae* was mostly comparable between the genotypes.

**Conclusion:**

Our findings demonstrate an important role of the adapter protein SLy2 in the context of adaptive antibody responses against pneumococcal conjugate‐vaccine. Interestingly, deficits in humoral immunity seemed to be compensated by cellular immune effectors upon bacterial challenge. Our study further shows a novel relevance of SLy2 for plasmablasts and B‐cell progenitors in the BM.

**Abbreviations**
BM: bone marrow
DS: Down syndrome
FO: follicular
GC: germinal center
Ig: immunoglobulin
IL: interleukin
Ko: knockout
LPS: lipopolysaccharide
MZ: marginal zone
P23: Pneumovax23
PCV13: Prevenar13
pPS: pneumococcal polysaccharide
SLy2: Src homology domain 3 lymphocyte protein 2
TD: thymus/T cell‐dependent
Tg: transgenic
TI: thymus/T cell‐independent
TLR: toll‐like receptor

## 1 INTRODUCTION

*Streptococcus pneumoniae* is an opportunistic pathogen in humans, asymptomatically colonizing the upper respiratory tract under normal conditions. Upon local spread, invasion of the lower airways or the bloodstream, *S. pneumoniae* can cause severe diseases such as sepsis, meningitis, otitis, and pneumonia.^1^, ^2^ It is the most common cause of community‐acquired pneumonia and mortality due to pneumococcal infections is especially high in infants, the elderly and in immunocompromised individuals.^3^ Despite the availability of vaccines, pneumococcal disease still constitutes a major global health problem.^2^, ^4^

Nowadays, two different types of pneumococcal immunizations are available. Pneumovax23 (P23) is a pure mixture of *S. pneumoniae*‐derived capsular polysaccharides and induces a thymus‐independent (TI) B‐cell response, relying on antibodies produced by innate B‐1 cells.^5^, ^6^ These cells are mainly located within the peritoneal and pleural body cavities but can also be found in the bone marrow (BM) and the spleen, where they constitutively secrete natural immunoglobulin M (IgM). B‐1 cell‐derived antibodies fulfill an important housekeeping function as they clear apoptotic cells and toxic metabolites from the circulation.^7^, ^8^ Besides, they quickly respond towards infections, thus representing an important first line defense against microbial pathogens, such as *S. pneumoniae*.^9^

The second vaccine is termed Prevenar13 (PCV13) and consists of 13 pneumococcal polysaccharides (pPS) coupled to a diphtheria carrier‐protein. Therefore, PCV13 accessorily induces thymus‐dependent (TD) responses that are mediated by adaptive B‐2 cells and provide long‐lived protection.^10^, ^11^ Since P23 has been shown to provide only transient and inadequate protection in high risk groups, the conjugate‐vaccine PCV13 is recommended for children less than 5 years, adults greater than 50 years and patients with immune defects.^5^

We recently reported a regulatory role of Src homology domain 3 lymphocyte protein 2 (SLy2), also termed HACS1 (hematopoietic adapter containing SH3 and SAM domains 1) or SAMSN1 (SAM domain, SH3 domain, and NLS 1), for B‐cell responses towards pneumococcal antigens.^12^, ^13^ SLy2 is an immunoinhibitory adapter protein that is mainly expressed in lymphocytes.^14^ It is a key player of intracellular signaling by recruiting and linking downstream effector molecules upon receptor‐induced activation. To this end, SLy2 holds specific protein interaction modules and is able to shuttle between the cytoplasm and the nucleus.^15^ In humans, SLy2 is encoded on chromosome 21q11.2, a region frequently disrupted by translocation events in the context of hematopoietic disorders.^16^ Several studies have highlighted the role of SLy2 for B‐cell responses.^12^, ^14^, ^17^, ^18^, ^19^ More precisely, it is upregulated in murine and human B cells upon activation.^18^

The investigation of SLy2‐deficient mice revealed an enlarged compartment of B‐1 cells and enhanced proliferative B‐cell responses in the absence of the adapter. Moreover, natural serum IgM levels are increased in these mice, accompanied by improved antibody responses to pPS.^13^, ^19^ On the contrary, SLy2‐transgenic (Tg) mice, specifically overexpressing the protein in T and B cells, harbor decreased proportions of B‐1 cells and significantly reduced titers of homeostatic IgM.^12^, ^17^ This was accompanied by impaired antibody responses towards the pPS‐vaccine P23.^12^ This is of high interest since SLy2 belongs into a group of 9 genes that are additionally amplified in Down syndrome (DS) patients and might contribute to the disease phenotype associated with Trisomy 21.^20^ DS patients suffer from several immune defects including abnormal antibody production and an enhanced susceptibility to pneumococcal infections.^21^, ^22^

Since TI B‐cell responses towards P23 are impaired in SLy2‐Tg mice, we were interested in their TD antibody production towards the conjugate‐vaccine PCV13, which is recommended for DS patients. Moreover, we evaluated the survival rate of SLy2‐Tg mice in comparison to their wildtype (Wt) littermates during acute pneumococcal infection. Our results revealed reduced rates of CD138^+^TACI^+^ plasmablasts (PBs) and PCV13‐specific immunoglobulin G (IgG) levels in SLy2‐Tg mice. We were unable to detect significant alterations with regard to the survival of SLy2‐Tg mice during acute pneumococcal lung infection, probably due to compensatory mechanisms. Interestingly, the numbers of BM B‐cell precursors were greatly diminished in SLy2‐Tg mice, which is of high interest as DS children also suffer from defects in B‐cell developmental processes.^22^, ^23^

## 2 MATERIALS AND METHODS

### 2.1 Mice

SLy2‐transgenic (Tg; over‐expressing) mice were generated as described previously and kept under specific pathogen‐free conditions.^17^ All animal work was performed according to the German animal care regulations and animal experiments were approved by the local ethics committee (AZ 29.03.2017; PH1/14 and PH2/19). For all experiments, age‐matched littermate mice were used. For PCV13‐vaccination studies, mice were 9–13 weeks old. For acute infection with *S. pneumoniae*, 16–17 weeks old mice were used.

### 2.2 Immunization experiments

Wt and SLy2‐Tg mice were intraperitoneally immunized with 3 µg Prevenar13 (Pfizer) in 100 µl phosphate‐buffered saline (PBS). To analyze cell populations and serum antibody titers, mice were sacrificed before and 7, 14, and 21 days after immunization to collect blood, peritoneal lavage (PL), spleen, and BM.

### 2.3 Organ preparation

Blood was collected in Microtainer blood collection tubes (BD Bioscience). After at least 30 min of incubation at RT, tubes were centrifuged at 15,000*g* for 90 s to collect the serum in the supernatant. Sera were stored at −20°C.

PL was performed with 5 ml of ice cold PBS. BM was collected by flushing out femurs with 5 mL of ice cold PBS. Spleens were homogenized with a 70 µm cell strainer and subsequently incubated in erythrocyte lysis buffer to get rid of red blood cells before analysis.

### 2.4 Pneumococcal infection

*S. pneumoniae* (ATCC strain 6303, serotype 3) was stored at −80°C in inoculated Roti‐Store Cryo tubes. The day before an infection, an inoculated ring was transferred into 5 ml sterile BHI medium and incubated at 37°C overnight without shaking. An OD_600_ of 0.5–0.7 defined a bacterial density of approximately 30 × 10^7^ colony forming unit (CFU)/ml in the original culture. Mice were slightly anesthetized and infected with either 1–2 (intermediate dose) or 2.5–3 (high dose) × 10^6^ CFU in 25 µl sterile PBS by intranasal application. Upon infection, mice were continuously monitored for 168 h. To estimate the degree of disease burden and to guarantee a consistent, well‐defined endpoint, weight, temperature, behavior, posture, and appearance of mice were assessed at least every 6 h during the first 3 days. If necessary, additional inspections during acute phase of infection took place every 3 h. Mice surviving the first 3 days of infection were subsequently controlled at least two times per day (more frequently if necessary), according to their health status and exactly as described above. Mice losing 15% of their starting weight or displaying a body temperature of less than 34.5°C were sacrificed immediately. One hundred and sixty‐eight hours after infection, all mice were sacrificed. For corresponding disease score data sheet please refer to supplementary Figure 1.

### 2.5 Enzyme‐linked immunosorbent assay

For assessment of specific immunoglobulin titers in the sera of mice, high‐binding plates were coated with 1 µg PCV13 in coating buffer overnight at 37°C. Before sample incubation, sera were diluted in sample buffer containing 10 µg/ml cell wall polysaccharides to capture unspecific antibodies. The samples were incubated on pre‐coated plates for 3 h at 37°C. Afterwards, biotinylated antimouse IgM, IgG_1_, IgG_2a_, or IgG_3_ antibody (BD Bioscience) were applied, followed by addition of streptavidin‐HRP conjugate (Biotechne). HRP‐reaction was induced with 3,3ʹ,5,5ʹ‐tetramethylbenzidine substrate (Thermo Scientific) and stopped by addition of sulfuric acid. Chemoluminescent read out was done at 450 out of 570 nm.

### 2.6 Cell culture

To analyze immune globulin class‐switch of B cells, CD19^+^ cells were isolated from the spleen by Magnetic Activated Cell Sorting using anti‐CD19 micro beads and magnetic separation columns (Miltenyi Biotech). Before stimulation, B cells were stained with the CellTrace CFSE Proliferation Kit (Invitrogen) at 37°C for 20 min and the reaction was stopped by addition of complete cell culture medium (RPMI medium supplemented with 10% FCS, 1% *l*‐glutamine, 1% penicillin/streptomycine (P/S) and 0.05 mM β‐mercaptoethanol). A total of 2 × 10^6^ B cells were cultured in 24‐well plate inserts in 500 µl medium either unstimulated, with 25 µg/ml lipopolysaccharide (LPS) only or with 25 µg/ml LPS plus 10 ng/ml interleukin‐4 (IL‐4) at 37°C and 5% CO_2_. After 48 h, cells and supernatants were analyzed via flow cytometry and via LEGENDplex, respectively.

### 2.7 LEGENDplex (multi‐analyte flow assay kit)

To determine the amounts of antibody secreted by activated B cells in culture, supernatants were analyzed using the LEGENDplex Mouse Immunoglobulin Isotyping Panel (BioLegend). The assay was performed in a V‐bottom plate according to manufacturer's protocol and data acquisition was done with the FACS Canto II Flow Cytometer (BD Bioscience). BioLegend's LEGENDplex Data Analysis Software was applied for analysis (www.biolegend.com/legendplex).

### 2.8 Flow cytometry

For flow cytometry, 1 × 10^6^ single cells from PL, BM, and spleen were incubated with anti‐mouse CD16/32 (BioLegend) for 15 min to block unspecific Fc‐binding sites. Subsequently, cells were fluorescently stained in two mixes to identify B‐1 cells, CD138^+^ cells, splenic B‐2 cells, and BM subpopulations: B220‐FITC, anti‐CD5‐PerCP‐Cy5.5, anti‐IgD‐V450, anti‐IgM‐APC‐Cy7, anti‐CD43‐PE, anti‐CD138‐APC and anti‐CD23‐BV510, anti‐B220‐PerCP, anti‐CD21‐FITC, anti‐CD23‐BV510, and GLY7‐PE (BioLegend and BD Bioscience). For identification of PBs and plasma cells (PCs), fluorescent staining was performed using anti‐B220‐FITC, anti‐CD19‐V450, anti‐TACI‐APC, anti‐CD138‐PE, and 7‐AAD for exclusion of dead cells.

To analyze surface immune globulins after in vitro stimulation, splenic B cells were incubated with anti‐CD19‐V450, anti‐CD43‐PE‐Cy7, anti‐CD5‐APC, anti‐IgM‐APC‐Cy7, anti‐IgD‐PerCP and anti‐IgG_1_‐PE (LPS/IL‐4) or with anti‐CD19‐APC‐Cy7, anti‐CD43‐PE, anti‐CD5‐APC, anti‐IgA‐BV421, anti‐IgG_2ab_‐BB700, and anti‐IgG_3_‐PE‐Cy7 (LPS only) (Biolegend and BD Bioscience). Proliferation was analyzed via carboxyfluorescein succinimidyl ester‐staining.

All measurements were performed at the BD FACS Canto II and biaxial gating was done with FlowJo Version 10. For flow cytometry gating strategies the reader is referred to Figure S2.

### 2.9 Statistics

All illustrations were designed with Graph Pad Prism Version 7. Statistical testing was done as indicated in corresponding figure legends.

## 3 RESULTS

### 3.1 Normal populations of adaptive B cells, but decreased rates of antibody‐secreting cells in SLy2‐Tg mice

Initially, we analyzed B‐1 cell populations of the peritoneum, spleen and the BM in SLy2‐Tg mice as compared to Wt littermates. As previously described by our group, the overexpression of SLy2 causes decreased proportions of innate B‐1 cells (Figure S3).^12^ The overall counts of splenocytes were comparable between the genotypes and adaptive B‐cell populations isolated from the spleen remained unaltered, including transitional T1 B cells, follicular (FO) B cells, marginal zone (MZ) B cells and germinal center (GC) B cells (Figure 1A). By contrast, the total number of BM cells was significantly reduced in SLy2‐Tg mice (Figure 1A).

Figure 1Frequency and count of B‐2 cell subsets and CD138^**+**^cells in the spleen and bone marrow (BM) of SLy2‐Tg and Wt control mice. (A) Graphs depict the total number of splenocytes and the frequency and number of CD138^+^ cells as well as following splenic B‐2 cell subsets: B220^+^CD23^‐^IgM^high^IgD^low^CD21^low^ transitional (T1) B cells, B220^+^CD23^+^IgM^+/−^ CD21^low^ follicular (FO) B cells, B220^+^CD21^+^IgM^+^CD23^−^ marginal zone (MZ) B cells, and B220^+^GLY7^+^ germinal center (GC) B cells. In addition, the total count of BM cells is shown together with BM‐resident CD138+ B‐cell populations. (B) Graphs depict the TACI and CD138 double‐positive cell populations of spleen and BM. These were further subdivided into B220^intermediate^ plasmablasts and B220^low^ plasma cells. Data were assessed via flow cytometry by analysis of *n* = 6–8 mice per genotype in two to three independent experiments. Error bars indicate the mean ± *SEM* and significance was determined by Student's *t* test with a *p* value of less than 0.05 considered statistically significant (*p < 0.05, **p < 0.01)

**Figure:**
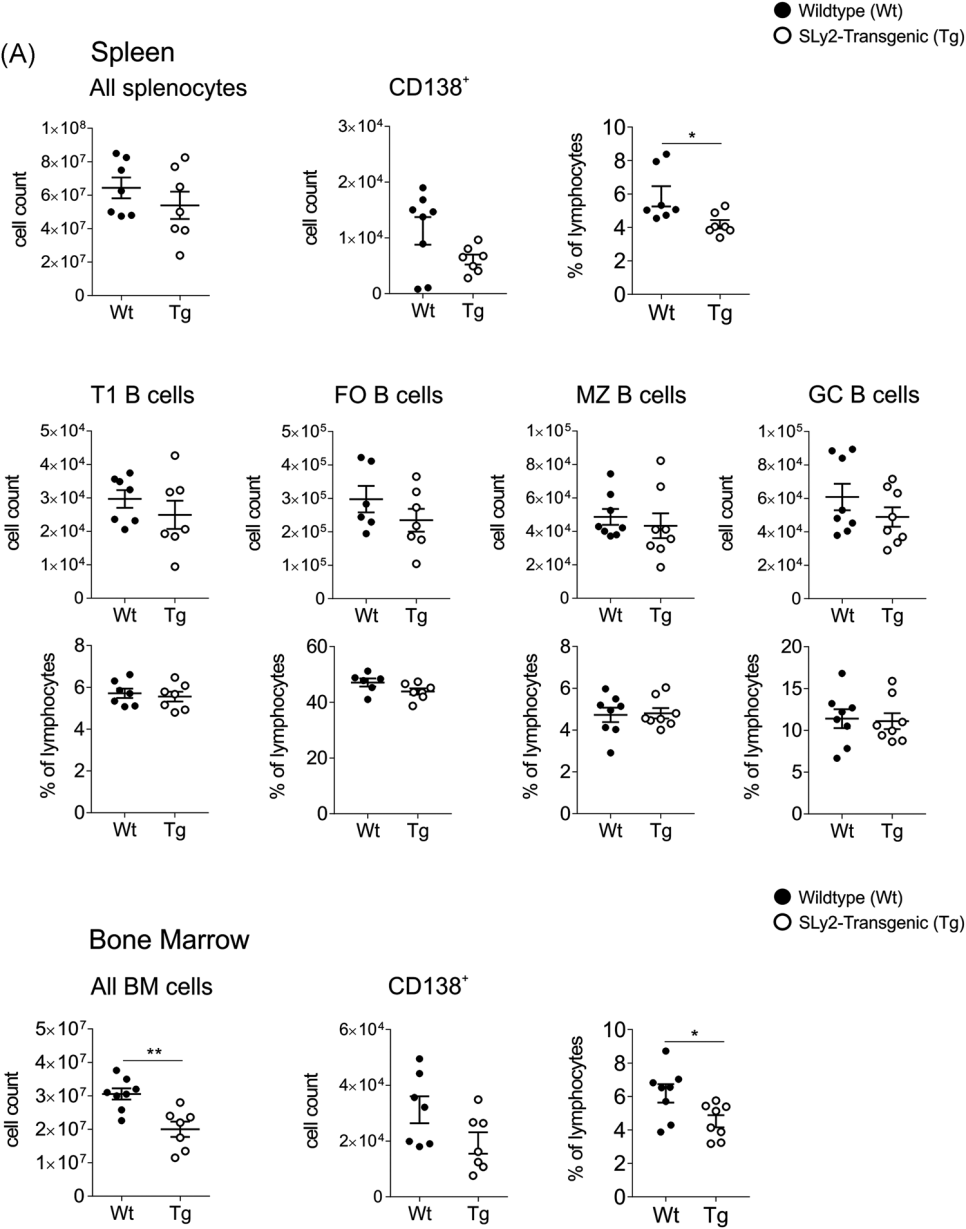

**Figure:**
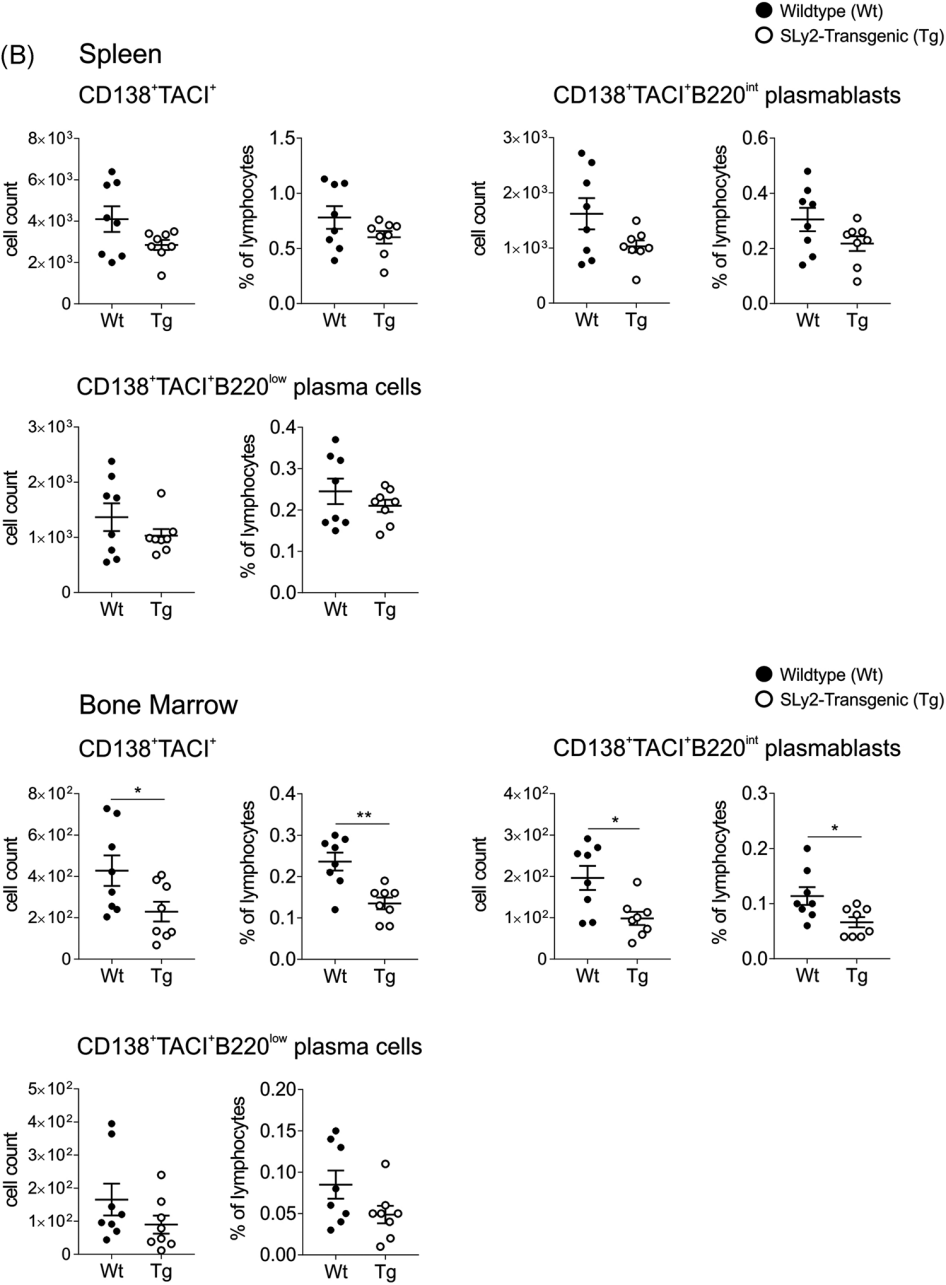

We recently reported decreased titers of serum IgM in SLy2‐Tg mice, indicating that excessive expression of the adapter protein impairs the antibody production in vivo.^12^ Since murine antibody‐secreting cells (ASCs) are known to express CD138 on their cell surface, we initially analyzed the proportion and number of CD138^+^ B cells in the spleen and BM of mice.^7^, ^24^ As shown in Figure 1A, the frequency of CD138^+^ cells was decreased in both organs of SLy2‐Tg mice.

Important to mention, CD138^+^ B cells constitute a heterogeneous population. For exact identification of PBs and PCs, additional markers, such as transmembrane activator and CAML interactor (TACI) are required.^25^ For this reason, we went into more detail by examining CD138 and TACI double‐positive cells, a subset that can be further subdivided into B220^intermediate^ PBs and B220^low^ PCs.^26^ As depicted in Figure 1B, PBs and PCs tended to be reduced in the spleen of transgenic mice; however these differences did not reach statistical significance. By contrast, the absolute number and frequency of CD138^+^TACI^+^ Cells were strongly diminished in the BM of SLy2‐Tg mice when compared to the Wt controls. The same applied to the specific fraction of CD138^+^TACI^+^B220^int^ PBs (Figure 1B). For flow cytometry gating strategies please refer to Figure S2.^26^

### 3.2 In vitro proliferation, class‐switch and antibody‐secretion of isolated B cells

**Figure 2:**
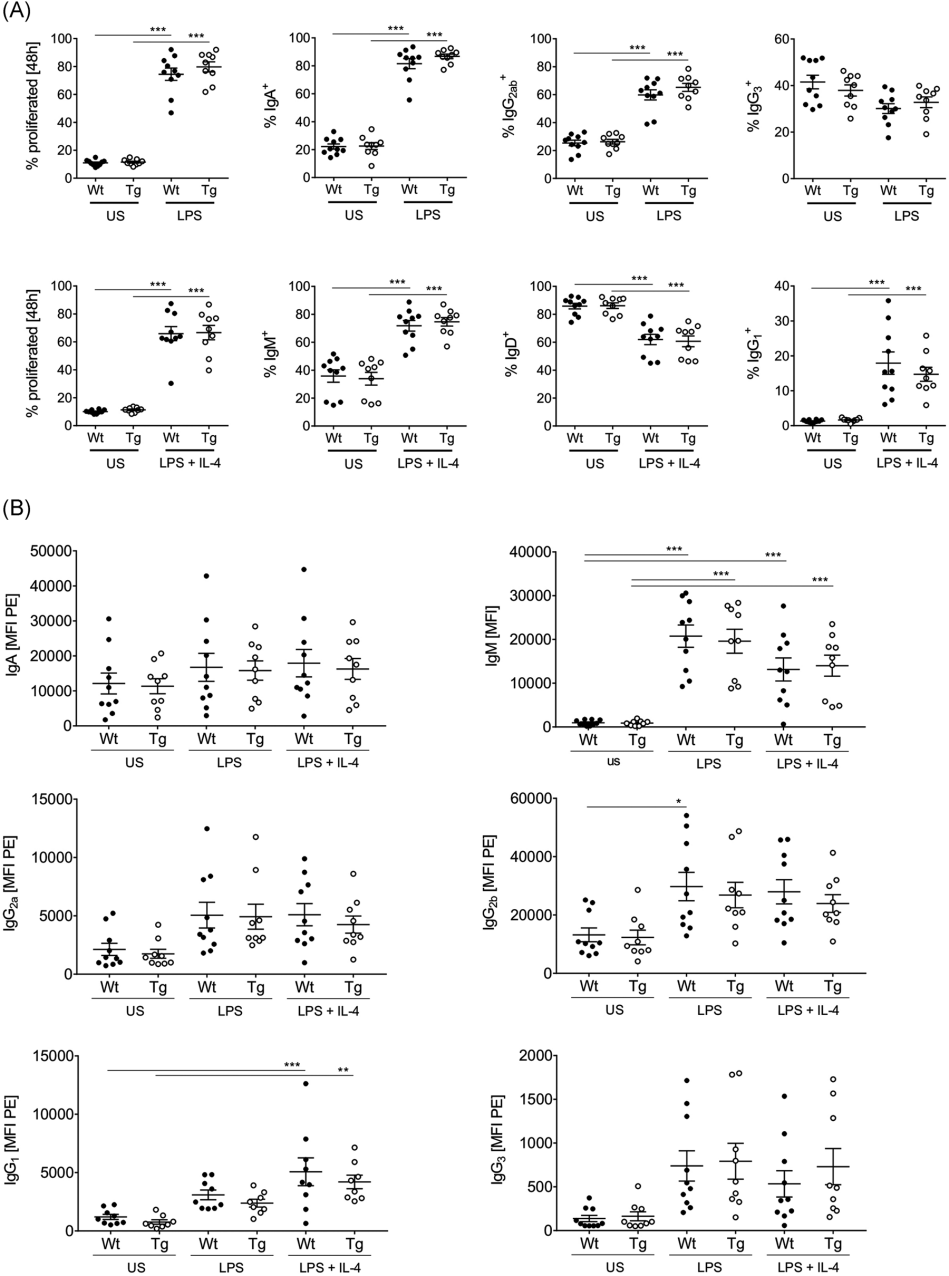
Surface immune globulin (Ig) class‐switch and antibody secretion of B cells upon in vitro stimulation. (A) The proliferation rate of isolated splenic B cells after 48 h of stimulation with lipopolysaccharide (LPS) only or LPS + interleukin‐4 (IL‐4) is shown. In addition, percentages of IgA^+^, IgG_2ab_^+^, IgG_3_^+^ (LPS, upper panel) and IgM^+^, IgD^+^, IgG_1_^+^ (LPS + IL‐4, lower panel) B cells are depicted. (B) After 48 h of stimulation, immune globulins in the cell culture supernatants were quantified by LEGENDplex analysis. Graphs depict the mean fluorescent index (MFI) of PE‐fluorescence, which corresponds to the concentration of either IgM, IgA, IgG_2a_, IgG_2b_, IgG_1_, or IgG_3_ in the supernatants. In every assay, an unstimulated (US) control was included. Data was assessed within three independent experiments using *n* = 5–9 mice per genotype. Significances were determined by one‐way analysis of variance (ANOVA) with a *p* value of less than 0.05 considered as statistically significant (*p < 0.05, **p < 0.01, ***p < 0.001). Error bars represent the mean ± *SEM*. IgG, immunoglobulin G; IgM, immunoglobulin M; Wt, wildtype

Since we found decreased ratios of ASCs in the spleen of SLy2‐Tg mice, we performed in vitro proliferation and class‐switch assays with isolated splenic B cells. Stimulation of CD19^+^ cells with LPS alone or LPS + IL‐4 significantly induced B‐cell proliferation (Figure 2A). In addition, LPS‐sensing triggered the upregulation of IgA and IgG_2ab_ on the surface of B cells on a highly comparable level in both groups (Figure 2A). Simultaneous supplementation of both stimulants led to a drastic reduction in IgD‐surface expression. Both, Wt and SLy2‐Tg B cells efficiently switched to IgG_1_ in response to LPS + IL‐4 (Figure 2A).

Consequently, we quantified the secreted immune globulins in the supernatants of the in vitro assays (Figure 2B). Both stimulation approaches induced high levels of secreted IgM and moderate levels of IgA, IgG_2ab_ and IgG_3_. Upon supplementation with IL‐4, the B cells released significant amounts of IgG_1_ into the supernatants (Figure 2B). There were no differences between the genotypes.

### 3.3 Impaired responses towards Prevenar13 in SLy2‐Tg mice

Our previous studies revealed a regulatory role of SLy2 for TI antibody responses towards the polysaccharide vaccine P23, as they were significantly impaired in its absence. This occurred in an IL‐5 reeceptor α (IL‐5Rα)‐dependent manner, since we could demonstrate decreased levels of IL‐5Rα surface expression on B‐1 cells.^12^

**Figure 3:**
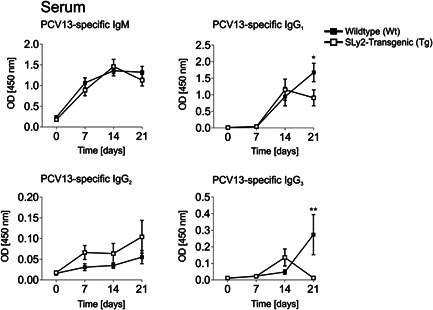
Prevenar13 (PCV13)‐specific immune globulin levels in the serum of mice. IgM, IgG_1_, IgG_2_, and IgG_3_ titers were determined by ELISA at Day 0 before immunization and 7, 14 and 21 days post‐immunization with PCV13. Graphs depict the optical density (OD) at 450 nm measurement wavelength over time (570 nm reference wavelength). All sera were incubated on one plate to allow for direct comparison of the time points. Curves represent n = 8‐11 mice per genotypes from two independently performed experiments. Error bars display the mean ± *SEM*. Significance was determined with two‐way ANOVA with a *p* value of less than 0.05 was considered statistically significant (*p < 0.05, **p < 0.01). ELISA, enzyme‐linked immunosorbent assay

We were therefore additionally interested in the TD antibody response of SLy2‐Tg mice towards the conjugate‐vaccine PCV13, which is recommended for high risk groups. As shown in Figure 3, PCV13‐specific IgM and IgG_2_ responses were comparable between the genotypes. However, 21 days post‐vaccination, Wt mice produced more amounts of IgG_1_ and IgG_3_ (Figure 3).

**Figure 4:**
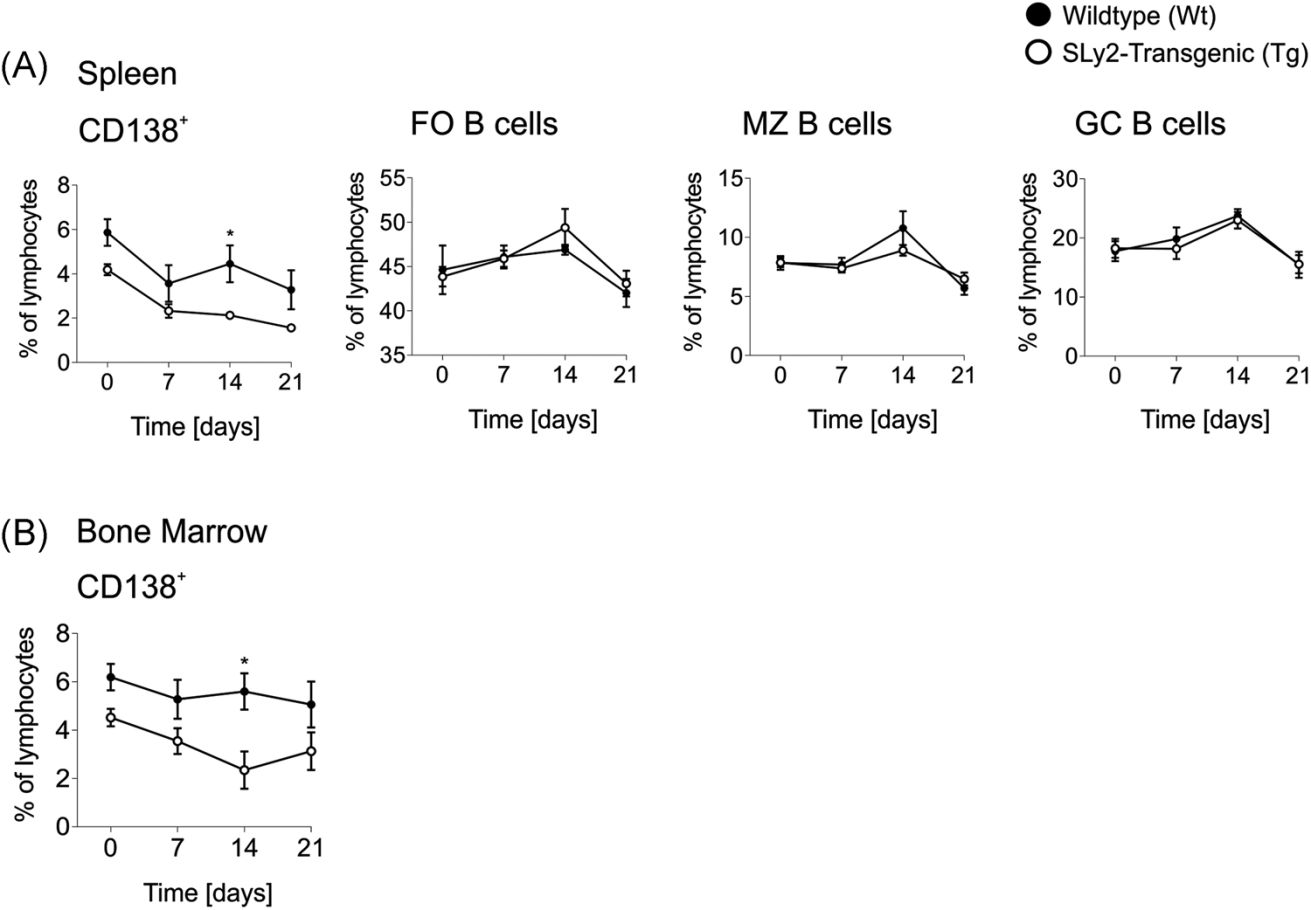
The over‐time progression of B‐cell populations upon vaccination with Prevenar13 (PCV13). Curves depict the over‐time progression of B‐cell frequencies within the pool of all lymphocytes as determined by flow cytometry. Populations shown are CD138^+^ cells, B220^+^CD23^+^IgM^+/−^ CD21^low^ FO, B220^+^CD21^+^IgM^+^CD23^−^ MZ B and B220^+^GLY7^+^ GC B cells in (A) the spleen and (B) the BM. Graphs represent *n* = 7–12 mice per genotype pooled from two or three independent experiments and error bars show the mean ± *SEM*. Significance was determined with two‐way ANOVA with a *p* value of les than 0.05 was considered statistically significant (*p < 0.05, ***p < 0.001). ANOVA, analysis of variance; BM, bone marrow; FO, follicular; GC, germinal center; IgM, immunoglobulin M; MZ, marginal zone

Accompanying, we investigated the progression of BM‐resident and splenic CD138^+^ cells as well as adaptive B‐2 cells in the spleen from Day 0 pre‐ to Day 21 post‐immunization. While splenic MZ B and B‐2 cell compartments were comparable, SLy2‐Tg mice significantly lacked CD138^+^ cells within the spleen and the BM at Day 14 post‐immunization (Figure 4).

### 3.4 Survival of SLy2‐Tg and Wt control mice in the course of acute pneumococcal infection

**Figure 5:**
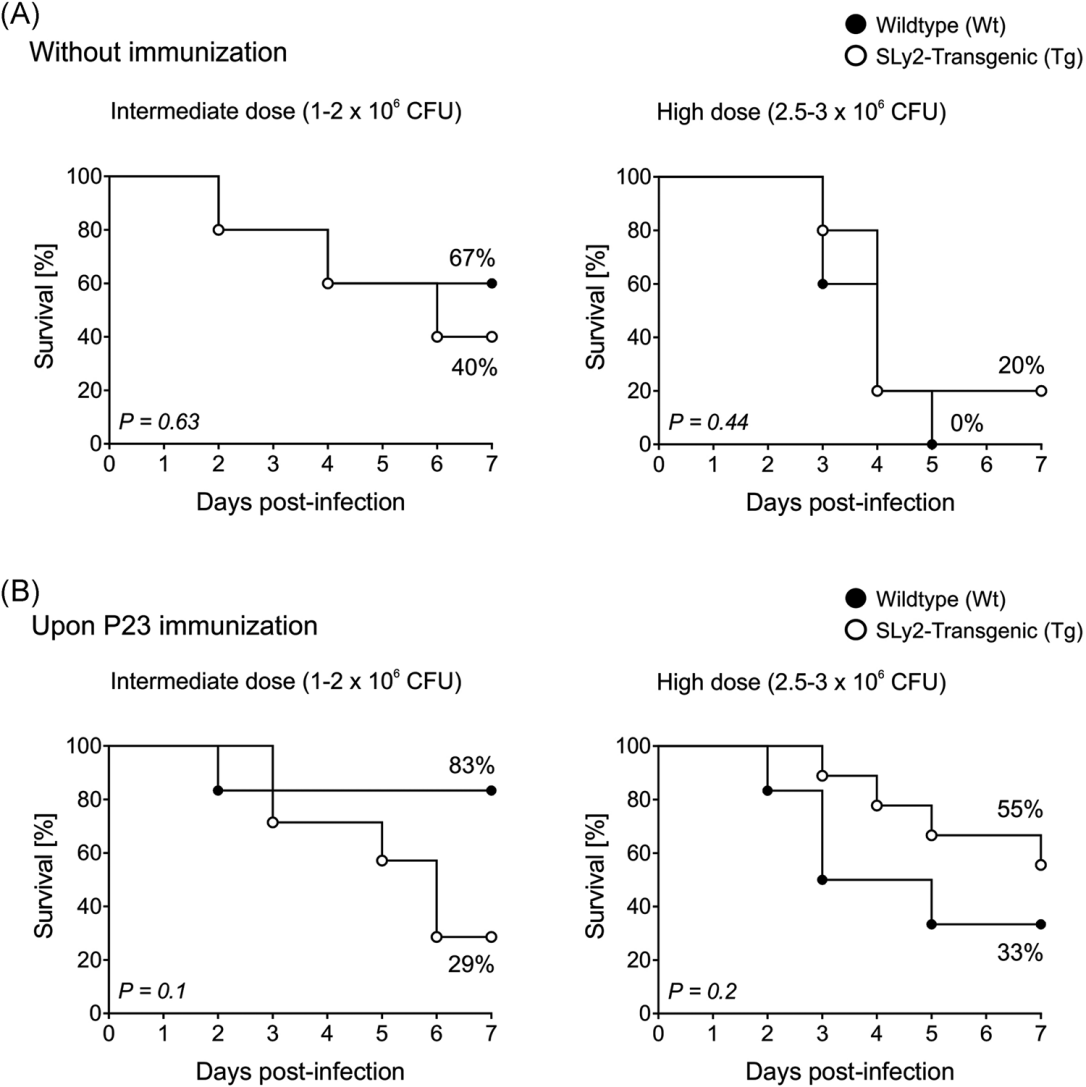
Survival analysis of SLy2‐Tg and Wt mice upon intranasal infection with *Streptococcus pneumoniae*. Mice were intranasally infected with either 1–2 × 10^6^ (intermediate) or 2.5–3 × 10^6^ (high) CFU of *S. pneumoniae* serotype 3 in 25 µL PBS. This was done (A) without preceding immunization or (B) 14 days after intraperitoneal vaccination with 1 µg P23. Each single survival curve represents *n* = 5–9 wildtype or SLy2‐transgenic mice as defined in the legends. Data is shown as Kaplan–Meier survival graph and indicated *p* values were determined by Log rank (Mantel Cox) test. CFU, colony forming unit

As SLy2‐Tg mice do not properly respond towards both, TI and TD pneumococcal vaccination, we next wanted to assess their survival rate during acute lung infection with *S. pneumoniae*. Thus, mice were infected without previous immunization (Figure 5A), as well as 14 days after injection of P23 (Figure 5B). For infection, we used two different doses of CFU: intermediate (1–2 × 10^6^ CFU/mouse) and high (2.5–3 × 10^6^ CFU/mouse).

Figure 5A depicts the survival curves of naïve mice after infection with the two varying doses of *S. pneumoniae*.

After exposure to an intermediate infection dose, 67% of all Wt mice and 40% of all SLy2‐Tg mice survived (Figure 5A, left panel). The challenge with a high dose of bacteria resulted in lower rates of survival, being 0% and 20% for Wt and SLy2‐Tg animals, respectively (Figure 5A, right panel). P23 vaccination before challenge conferred protection to Wt mice, as 83% of them survived the intermediate dose (Figure 5B, left panel) and 33% the high dose (Figure 5B, right panel). Surprisingly, SLy2‐Tg animals infected with an intermediate dose of *S. pneumoniae* did not benefit from preceding immunization, as only 29% of them survived (Figure 5B, left panel). By contrast, upon administration of a high infection dose, SLy2‐Tg mice were protected as well, with their survival rate being 55% (Figure 5B, right panel). There were no statistically significant differences in survival between the genotypes in all conditions examined.

### 3.5 Decreased counts and proportions of B‐cell precursors in the bone marrow of SLy2‐Tg mice

Classically, B‐2 cells develop within the BM from hematopoietic precursors, thereby undergoing a range of selection and differentiation steps. Meanwhile, they individually rearrange their immune globulin gene loci and finally express a unique B‐cell receptor on their surface to leave the BM as mature naïve B cells.^27^, ^28^ While B‐1a cells mainly derive from the fetal liver early in ontogeny, B‐1b cells and adaptive B‐2 cells can be reconstituted from BM precursors.^29^, ^30^, ^31^, ^32^

**Figure 6:**
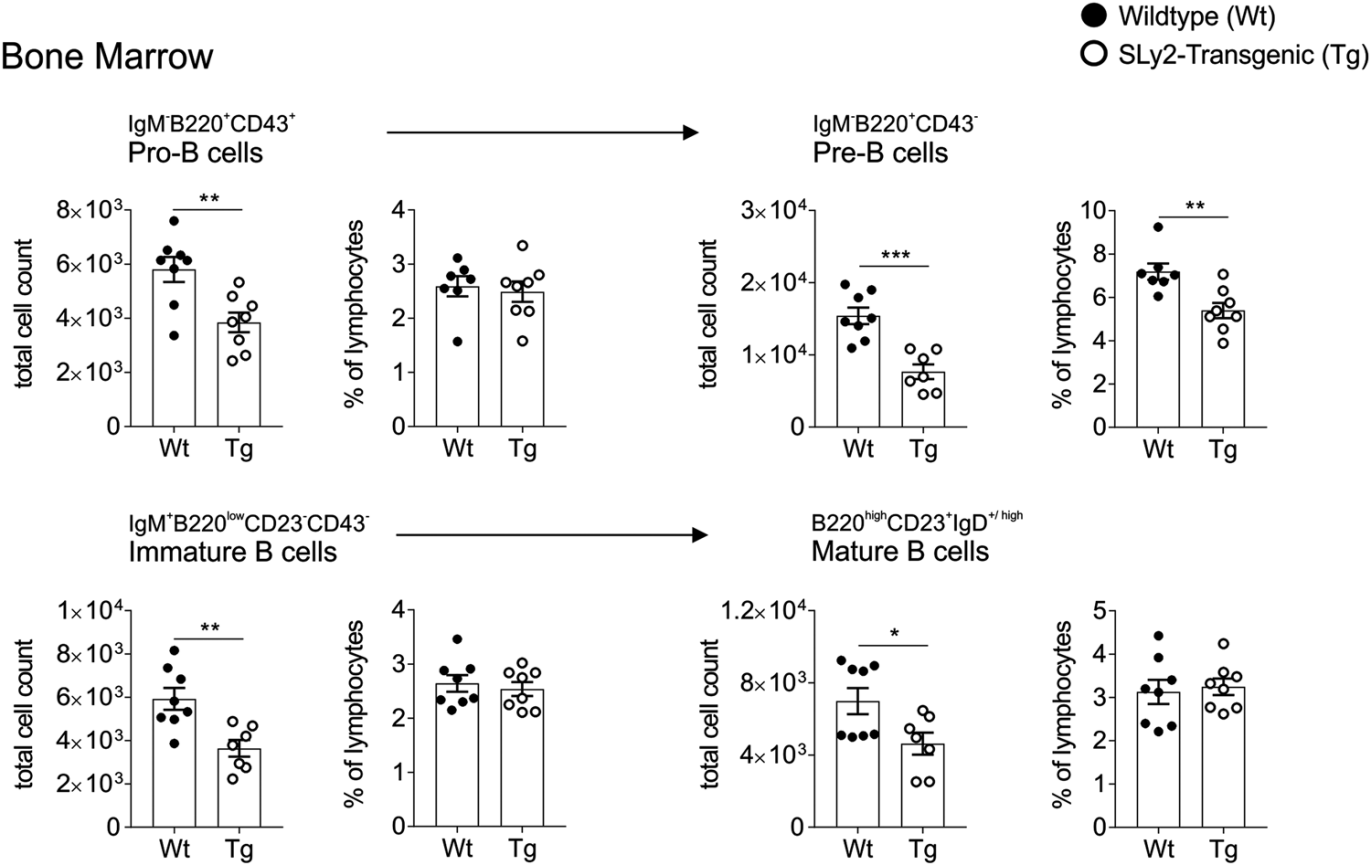
B‐cell developmental stages within the BM of SLy2‐Tg and Wt littermate mice. B‐cell precursors of the BM were investigated by flow cytometry. The percentage within the overall lymphocyte population and the total count of cells are shown for IgM^−^B220^+^CD43^+^ Pro‐B cells, IgM^‐^B220^+^CD43^−^ Pre‐B cells, IgM^+^B220^low^CD23^−^ immature B cells and B220^high^CD23^+^IgD^+^ mature B cells. Data represent *n* = 7–8 mice per genotype from two independent stainings. Error bars indicate the mean ± *SEM* and significance was determined by Student's *t* test with a *p* value of less than 0.05 considered statistically significant (*p < 0.05, **p < 0.01, ***p < 0.001). BM, bone marrow; IgM, immunoglobulin M; Wt, wildtype

Since the total number of BM cells was drastically reduced in SLy2‐Tg mice, we next analyzed different precursor populations according to their surface marker expression. As shown in Figure 6, almost all stages of B‐cell progenitors were impaired upon overexpression of SLy2. More precisely, the total cell counts of pro‐B cells, pre‐B cells as well as immature and mature B cells were significantly reduced (Figure 6). In addition, pre‐B cells were also decreased in their ratio within the overall lymphocyte population.

## 4 DISCUSSION

Our previous report on SLy2‐Tg mice demonstrated decreased populations of innate B‐1 cells and attenuated titers of natural antibodies caused by lymphocyte‐specific overexpression of the adapter protein SLy2. This was accompanied by a significantly impaired TI response to the pure polysaccharide vaccine P23.^12^ In the present work, we aimed to investigate TD responses towards the conjugate‐vaccine PCV13 in the context of excessive SLy2‐expression, with an emphasis on adaptive B‐cell subsets.

TD protein antigens usually trigger a rapid extrafollicular response, shortly followed by the formation of a GC reaction within the B‐cell follicles.^33^, ^34^ The GC is a highly dynamic process during which FO B cells undergo somatic hypermutation and are positively selected for increasing antigen affinity. This occurs in dependence of specialized cognate T follicular helper (T_FH_) cells. Eventually, highly specific effector B cells arise, including class‐switched plasma cells and long‐lived memory B cells.^35^, ^36^ Classically, the immune globulin isotypes evoked during GC reaction are IgG_1_ and IgG_3_.^37^, ^38^

Conveniently, injection of PCV13 triggered huge amounts of specific serum IgM, followed by IgG_1_ and IgG_3_ after 2 weeks (Figure 3). Surprisingly, the IgM serum concentrations were fully comparable within the genotypes, indicating that SLy2 controls IgM induction under TI, but not TD conditions (Figure 3). This is in accordance with recent findings regarding SLy2‐deficient mice.^13^ By contrast, SLy2‐Tg mice produced significantly lower amounts of IgG_1_ and IgG_3_ than their Wt counterparts. More precisely, while PCV13‐directed IgG_1_ and IgG_3_ levels peaked in Wt mice at Day 21 post‐immunization, they already dropped after 14 days in the sera of transgenic animals (Figure 3).

The observation that SLy2‐Tg mice still generated equal levels of IgG_1_ and IgG_3_ at Day 14 post‐vaccination points to normal induction of GC reaction and class‐switch in these mice. This thought is strongly supported by the fact that in vitro class‐switch and Ig secretion of B cells were fully comparable between the genotypes (Figure 2A,B). Moreover, this points to a regulatory role of the adapter protein SLy2 specifically in the context of antibody responses to *S. pneumoniae*‐derived polysaccharides.

The populations of splenic FO and GC B cells were unaltered in SLy2‐Tg mice; both pre‐ and post‐immunization (Figures 1 and 4). These results indicate that the overexpression of SLy2 might specifically impair extrafollicular TD responses.^39^ On the other hand, the reduced serum IgG_1_ and IgG_3_ titers were preceded by markedly decreased ratios of splenic and BM‐resident CD138^+^ cells at Day 14 in the context of SLy2‐overexpression (Figure 4). In addition, if compared to Wt littermates, these mice significantly lacked steady state CD138^+^TACI^+^B220^int^ PBs (Figure 1B). Notably, further experiments are required to clarify whether these PBs are mainly derived from the B‐1 or the B‐2 cell compartment.

Collectively, these findings point to a possible implication of the adapter protein SLy2 in ASC differentiation and/or survival. It is well‐established, that the initiation of the genetic ASC program depends on Blimp‐1 mediated gene repression.^40^, ^41^ Hence, one possibility would be a SLy2‐mediated repression of Blimp‐1 and as a consequence, inhibition of ASC development. On the other hand, XBP‐1 is a master regulator of ASC activity, by driving the expression of Ig transcripts in plasma cells.^42^ Thus, SLy2 could as well be implicated in XBP‐1 mediated regulation of antibody production. Based on our findings, the role of SLy2 in ASC‐specific signaling pathways should be subject to subsequent work.

Of interest, we recently reported improved PCV13‐specifc IgG_2_ responses in mice deficient for SLy2 and thus conclude, that the overexpression of the adapter protein does not necessarily reflect the exact opposite of its homozygous deletion.^13^ Nevertheless, these data collectively demonstrate that SLy2 controls both, the generation of specific IgM and IgG towards TI and TD pneumococcal vaccine, respectively.

Surprisingly, despite the impaired antibody response of SLy2‐Tg mice towards both pure pPS and conjugate‐vaccine, they had no statistically significant survival disadvantage upon intranasal infection with *S. pneumoniae*. While SLy2‐Tg mice tended to be more prone towards infection with intermediate units of bacteria (Figure 5B, left panel), they even displayed a better survival rate in the context of a high infection dose (Figure 5B, right panel). Immune responses to *S. pneumoniae* always involve both, humoral and cellular components: pPS‐ and protein‐specific antibodies act as neutralizing and opsonizing agents, while CD4^+^ T_H_ cells rapidly recruit phagocytes towards the airways by producing inflammatory cytokines.^43^ While antibodies have been shown to be of upmost importance regarding septicemia, the response to pneumococcal colonization in the nasopharynx seems to solely depend on IL‐17‐secreting CD4^+^ T_H_ cells. More precisely, the mucosal clearance of pneumococcal antigens upon intranasal application was fully functional in a study on µMT mice, lacking B cells and antibodies.^44^ By contrast, immunity to septicemia absolutely required CD138^+^ ASCs.^43^, ^45^ Based on this knowledge we hypothesize, that cellular components of the immune system compensated for the lack of antibodies in SLy2‐Tg mice in our murine model of pneumonia, especially after facing a high infection dose (Figure 5). Previous experiments with SLy2‐deficient mice further support this hypothesis, as these did not benefit from their increased serum antibody titers during pneumonia.^13^ In addition, when assessing the bacterial burden in the blood, we found that our infection model was exclusively restricted to the lung (data not shown). We therefore plan to investigate the role of SLy2 during pneumococcal sepsis by intravenous infection of our mice.

The family of SLy/SASH‐adapter proteins comprises three highly homologous members, namely SLy1, SLy2, and SLy3/SASH1.^46^, ^47^ Apart from SLy2, lymphocytes express high levels of SLy1, which has been shown to be indispensible for normal development of thymocytes.^47^, ^48^

In the present work, we found greatly reduced numbers of B‐cell precursors in the BM of SLy2‐Tg mice as compared to Wt littermates (Figure 6). Importantly, this deficit applied to all stages examined, including pro‐B, pre‐B and immature B cells. This indicates that SLy2 not only interferes with one specific transition stage, but affects the overall progenitor population (Figure 6). B‐cell development is a multistep process. Initially, hematopoietic stem cells give rise to common lymphoid progenitors, which are committed to the lymphocyte lineage.^49^ Subsequently, the B‐cell developmental program is driven by tight regulation of specific transcription factors. For example, the differentiation of pro‐B and pre‐B cells is controlled by the master regulator Pax‐5.^50^ Pax‐5 is expressed throughout B lymphopoiesis and its inactivation leads to a reversion towards an uncommitted precursor state.^51^ Negative regulation of Pax‐5‐expression by SLy2 could be one possible explanation for the severely reduced B‐cell precursors in SLy2‐Tg mice, hence this should be subject to future studies.

Interestingly, previous studies on mice expressing a mutated version of the highly homologous protein SLy1 revealed an impaired ability of the hematopoietic system to reconstitute the lymphocyte compartment.^47^ Further, novel data assessed by our group demonstrate a role of the SLy1‐protein downstream of the IL‐7R and preTCR in developing thymocytes (unpublished data). While the percentages of pro‐B cells and immature B cells were unaltered in SLy2‐Tg mice, the proportion of pre‐B cells within the overall lymphocyte population was diminished (Figure 6). This observation could point to a special role of SLy2 in pro‐B to pre‐B transition, which among others depends on the IL‐7 signaling pathway.^52^ Based on the high sequence similarity and conservation of the SLy/SASH‐proteins, SLy2 should be considered as a potential player during IL‐7/IL‐7R‐signaling in developing B cells. Collectively, these findings allow speculating that SLy2 might take over similar roles in lymphocyte development as does its homolog SLy1.

Of note, since the SLy2‐transgene in our mouse model is expressed in both B and T cells, an additive inhibition of early T‐cell progenitors should be debate of discussion; especially since our phenotype investigation did not include the CD19‐marker (Figure 6). However, in B cells, the SLy2‐transgene is under the control of the Eµ IgH enhancer and therefore already expressed in early pro‐B cells, rearranging their heavy chain.^53^ By contrast, T‐cell specific SLy2‐overexpression depends on the *lck*‐promoter, which is initially activated in thymocytes.^54^ Thus, our observations are likely to reflect a specific effect of excessively expressed SLy2 on developing B cells.

By interfering with B lymphopoiesis, SLy2 might negatively control the reconstitution of certain B‐cell compartments in SLy2‐Tg mice. This is interesting since SLy2 is significantly overexpressed in DS patients, which mount inadequate antibody responses to vaccination and suffer from B lymphocytopenia.^22^, ^55^ More precisely, fetuses with DS show a significant decrease in CD19^+^ lymphocytes. Furthermore, while B cells usually undergo enormous expansion during the first year of life, this process was shown to be defective in children with DS when compared to healthy controls.^23^, ^56^ Hence, follow‐up studies should focus on whether SLy2 is implicated in transcriptional regulation and key pathways during B‐cell development. In this regard, Pax‐5 and the IL‐7‐signaling pathway might be promising targets.

## 5 CONCLUSION

In summary, the present study reveals impaired antibody responses to pneumococcal conjugate‐vaccine in SLy2‐Tg mice, accompanied by decreased populations of BM‐resident plasmablasts. The overall survival rate upon acute pneumococcal lung infection was comparable between the genotypes, indicating that the impaired antibody‐mediated protection in SLy2‐Tg mice could be compensated by cellular effectors. Besides, our findings demonstrate a novel role of the immunoinhibitory adapter protein SLy2 for B‐cell developmental processes, as reflected by severely reduced numbers of precursor cells within the BM.

## CONFLICT OF INTERESTS

The authors declare that there are no conflict of interests.

## AUTHOR CONTRIBUTIONS

Jennifer Jaufmann designed and carried out the experiments, analyzed the data and wrote the manuscript. Leyla Tümen performed experiments and analyzed the data. Sandra Beer‐Hammer designed experiments, provided experimental and conceptual advice and wrote the manuscript.

## Supporting information

Supporting information.

Supporting information.

Supporting information.

Supporting information.

## Data Availability

The data that support the findings of this study are available from the corresponding author upon reasonable request.
