# Supplementary material for: SLy2‐overexpression impairs B‐cell development in the bone marrow and the IgG response towards pneumococcal conjugate‐vaccine
Source: Immun Inflamm Dis. 2021 Feb 16;9(2):533–46. doi: 10.1002/iid3.413 (PMC8127564; doi:10.1002/iid3.413)
Supplement: Supplementary file 1 — Supporting information. [file IID3-9-533-s003.pdf]

Disease score sheet

| Parameter | Observation | Score |
|---|---|---|
| ***Alterations in body weight***<br><br>*(corrected according to the age-dependent weight gain that has to be expected)* | *No alterations* | *0* |
| | *5-10% weight loss as compared to the starting weight one day prior to infection* | *1* |
| | *10%-14% weight loss as compared to the starting weight one day prior to infection* | *2* |
| | *≥ 15% weight loss as compared to the starting weight one day prior to infection* | *Instant termination of the experiment and euthanasia* |
| ***Fur*** | *normal, shiny* | *0* |
| | *scruffy* | *1* |
| | *dirty/ diarrhoea* | *2* |
| ***Breathing*** | *normal* | *0* |
| | *flat* | *1* |
| | *heavy, fast and abdominal* | *Instant termination of the experiment and euthanasia* |
| ***Posture*** | *normal* | *0* |
| | *curvy or slightly bent* | *1* |
| | *heavily bent* | *Instant termination of the experiment and euthanasia* |
| ***Temperature (rectal)*** | *≤ 34.5°C* | *Instant termination of the experiment and euthanasia* |

***Disease Score = 4***
→ *Instant termination of the experiment and euthanasia*

Supplementary Figure 1
