# Supplementary figures and images for: SLy2‐overexpression impairs B‐cell development in the bone marrow and the IgG response towards pneumococcal conjugate‐vaccine
Source: Immun Inflamm Dis. 2021 Feb 16;9(2):533–46. doi: 10.1002/iid3.413 (PMC8127564; doi:10.1002/iid3.413)

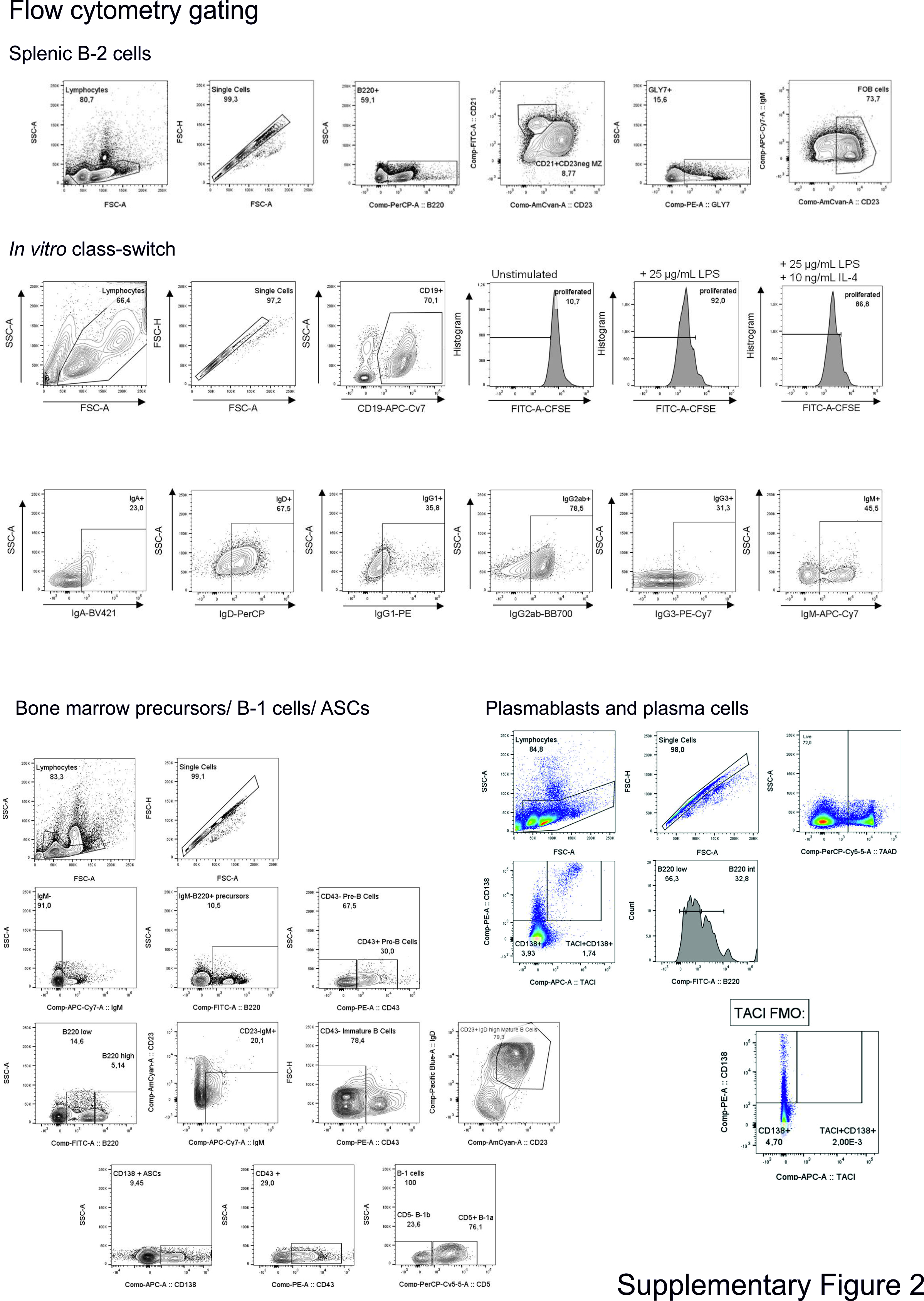

Supplement: Supplementary file 2 — Supporting information. [file IID3-9-533-s001.jpg]

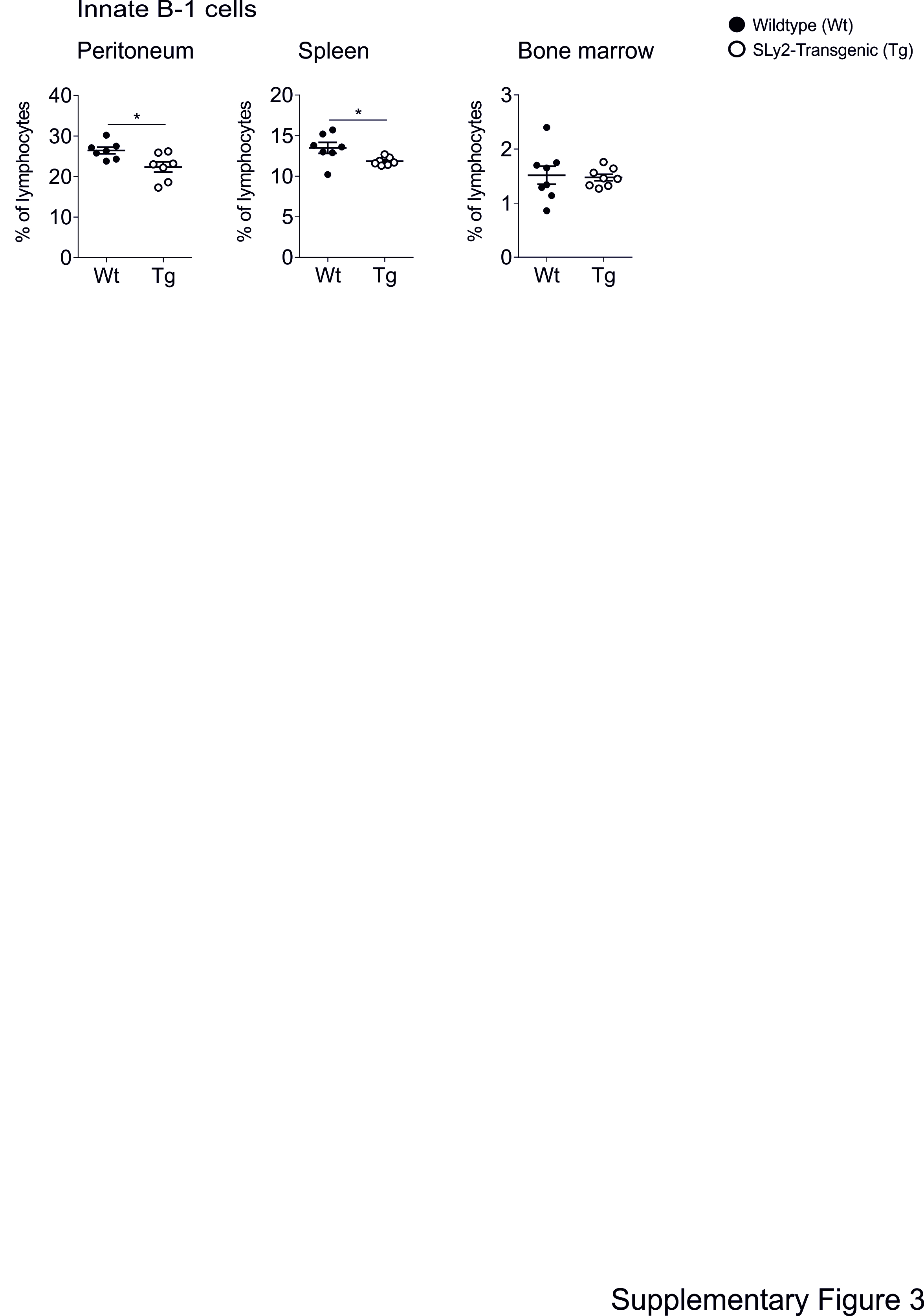

Supplement: Supplementary file 3 — Supporting information. [file IID3-9-533-s004.jpg]
