# Supplementary material for: SLy2‐overexpression impairs B‐cell development in the bone marrow and the IgG response towards pneumococcal conjugate‐vaccine
Source: Immun Inflamm Dis. 2021 Feb 16;9(2):533–46. doi: 10.1002/iid3.413 (PMC8127564; doi:10.1002/iid3.413)
Supplement: Supplementary file 4 — Supporting information. [file IID3-9-533-s002.docx]

Supplementary Figure Legends

**Supplementary Figure 1: Disease score sheet.** Murine health parameters that were considered to estimate the degree of disease burden during acute pneumococcal infection are listed. According to these health parameters, the disease score of the mice was constantly monitored in detail. A disease score of 4, a weight loss of ≥ 15% or a rectal temperature of ≤ 34.5°C were defined as immediate endpoints.

**Supplementary Figure 2: Flow cytometry gating.** The gating strategies applied for identification of all B-cell subsets are exemplarily shown.

**Supplementary Figure 3: Innate B-1 cell populations in SLy2-Tg and Wt animals.** Graphs depict B220^low^CD43^+^IgM^+^ B-1 cells in the peritoneum, spleen and BM of SLy2-Tg and Wt mice at steady state. Data represent n=7-8 mice per genotype from two independent flow cytometry experiments and the error bars display the mean ± SEM. Significance was determined by *Student’s t-test* with a p-value <0.05 considered statistically significant (*p<0.05, **p<0.01).
